# Global-Scale Metabolomic Profiling of Human Hair for Simultaneous Monitoring of Endogenous Metabolome, Short- and Long-Term Exposome

**DOI:** 10.3389/fchem.2021.674265

**Published:** 2021-05-12

**Authors:** Ying Chen, Jian Guo, Shipei Xing, Huaxu Yu, Tao Huan

**Affiliations:** Department of Chemistry, Faculty of Science, University of British Columbia, Vancouver, BC, Canada

**Keywords:** liquid chromatography-mass spectrometry, hair metabolomics, exposome, endogenous metabolites, short-term exposures, long-term exposure

## Abstract

Hair is a unique biological matrix that adsorbs short-term exposures (e. g., environmental contaminants and personal care products) on its surface and also embeds endogenous metabolites and long-term exposures in its matrix. In this work, we developed an untargeted metabolomics workflow to profile both temporal exposure chemicals and endogenous metabolites in the same hair sample. This analytical workflow begins with the extraction of short-term exposures from hair surfaces through washing. Further development of mechanical homogenization extracts endogenous metabolites and long-term exposures from the cleaned hair. Both solutions of hair wash and hair extract were analyzed using ultra-high-performance liquid chromatography-high-resolution mass spectrometry (UHPLC-HRMS)-based metabolomics for global-scale metabolic profiling. After analysis, raw data were processed using bioinformatic programs recently developed specifically for exposome research. Using optimized experimental conditions, we detected a total of 10,005 and 9,584 metabolic features from hair wash and extraction samples, respectively. Among them, 274 and 276 features can be definitively confirmed by MS^2^ spectral matching against spectral library, and an additional 3,356 and 3,079 features were tentatively confirmed as biotransformation metabolites. To demonstrate the performance of our hair metabolomics, we collected hair samples from three female volunteers and tested their hair metabolic changes before and after a 2-day exposure exercise. Our results show that 645 features from wash and 89 features from extract were significantly changed from the 2-day exposure. Altogether, this work provides a novel analytical approach to study the hair metabolome and exposome at a global scale, which can be implemented in a wide range of biological applications for a deeper understanding of the impact of environmental and genetic factors on human health.

## Introduction

The development of ultra-high-performance liquid chromatography-high-resolution mass spectrometry (UHPLC-HRMS) has enabled large-scale, unbiased profiling of metabolites in a given biological system (Patti et al., 2012; Johnson et al., 2016). LC-MS-based metabolomics has achieved great success in the post-genomic era of biology, including but not limited to the discovery of disease biomarkers and understanding of biological mechanisms (Gimple et al., 2019; Li et al., 2020). In particular, the rapid development of LC-MS-based global metabolomics (Creek et al., 2011; Vuckovic, 2012; Doerr, 2017) has stimulated the study of the exposome, which is defined as the totality of environmental exposures that an individual experiences over a lifetime (Wild, 2005; Warth et al., 2017). Currently, exposome research is mostly carried out using blood and urine samples, both of which are ideal biological matrices that contain endogenous metabolites produced by the host as well as exogenous metabolites taken in from the environment, such as diet and drug use. These biological matrices excel in reflecting temporal exposure, suggesting health condition, and dictating immediate intoxication status (Byard and Payne-James, 2015).

However, blood and urine-based exposome research is not without its limitations. Since blood and urine are considered biological hazards and contain bioactive enzymes and bacteria, their sample collection needs to be performed with extra caution and usually requires critical storage conditions (i.e., −80° freezer). In comparison to blood and urine, hair offers several potential advantages for exposome research. First of all, hair samples can be collected with non-invasive procedures and are conveniently stored and transported. Secondly, hair can retain both long- (days to months) and short-term (within 24 h) exposome information (Al-Delaimy, 2002; Sauvé et al., 2007). For instance, the accumulation of heavy metals and environmental pollutants in hair are well above those levels present in blood or urine, making it a suitable diagnostic tool for the history of an individual's exposures. Last but not least, the biochemical composition of hair can reveal a person's age, sex, smoking habits, and hair cosmetics (Chojnacka et al., 2006). Therefore, hair has been recognized for many decades as an ideal biological matrix and a valuable source of information in scientific research (Finner, 2013), clinical diagnosis (Klevay et al., 1987; Kintz et al., 2015), and forensic analysis (Moeller et al., 1993; Tagliaro et al., 1998).

However, in most of these works, the analytical assays are designed to target one or a panel of chemicals that are critical to the given analytical and bioanalytical questions. Despite the resounding importance of hair in metabolomics-guided understanding environmental exposures, it has not been widely used in exposome research compared to blood and urine. This is related to both the analytical and bioinformatic challenges for hair-based exposome research. First, hair is an extremely harsh matrix; extracting metabolites from hair is much more challenging compared to extracting metabolites from blood and urine. Conventional approaches of hair digestion using acid and base hydrolysis followed by heating are not fit for the purpose of omics-scale untargeted profiling as many metabolites are pH sensitive and thermolabile (Robbins and Robbins, 2012; Sulek et al., 2014). In addition, endogenous metabolites and exposure chemicals present diverse chemical structures with wide concentration ranges. Many of the features do not have good peak shapes and are difficult to identify using conventional peak-fitting based feature extraction algorithms (Smith et al., 2006; Hu et al., 2019). Moreover, many exposure chemicals have not been reported in literature, and their chemical identities are completely unknown (i.e., the “unknown unknown”; Stein, 2012). This makes it extremely difficult to perform annotation to understand and interpret MS-based exposome data.

In this work, we explored the possibility of using hair samples for exposome research by incorporating state-of-the-art analytical and bioinformatic developments in MS-based metabolomics. To achieve this goal, we developed an untargeted workflow to extract short-term exposure as well as long-term exposure and endogenous metabolites. Orthogonal LC-MS analyses were performed to detect and quantify a wide range of metabolites. To effectively process LC-MS data, we applied a recently developed integrated feature extraction algorithm to capture both high and low abundant metabolic features (Hu et al., 2019), followed by metabolite annotation using McSearch to capture novel metabolites that are not archived in a spectral library (Xing et al., 2020). For data interpretation, we manually sourced and resolved the chemicals on hair surfaces and in hair strands by searching against the latest version of Human Metabolome Database (HMDB; Wishart et al., 2018). Our results show that endogenous metabolites were dominant in hair extract, while metabolites from environmental contaminants and personal care products dominated in hair wash. As a demonstration of our hair metabolomics workflow, hair samples from three volunteers were collected before (immediately after regular hair washing) and after a 48-h exposure activity. The results of hair washes from before and after exposure were compared to indicate the effect of environmental factors on hair metabolomics. Hair extracts from before and after exposure were also compared, suggesting that metabolites firmly attached to the hair shaft were barely affected by the environment and the wash process.

## Materials and Methods

### Chemicals and Materials

LC-MS grade water (H_2_O), acetonitrile (ACN), methanol (MeOH), acetone (ACE), and other solvents were purchased from Fisher Scientific (Hampton, New Hampshire). All buffer reagents and other chemicals were purchased from Sigma-Aldrich (St. Louis, Missouri). XXTuff Reinforced vials and chrome steel beads were purchased from Biospec (Bartlesville, Oklahoma).

### Hair Sampling

The hair sampling protocol was adapted from the Society of Hair Testing guidelines for drug testing in hair (Cooper et al., 2012). Scissors and hair clips were first cleaned with alcohol wipes. Donor's hair was parted horizontally across the crown and was secured out of the way with a hair clip. Approximately 30–40 strands of hair were cut from five small areas along the part line to avoid bald patches. Collected hair strands were aliquoted into three portions (three replicates). The hair sample was cut as close to the scalp as possible. Collected samples were stored at −80°C prior to metabolomics experiments. The human ethics of hair collection protocol (H19-01825) has been approved by University of British Columbia.

### Vial and Bead Cleaning

To minimize ion suppression caused by contaminants, sample vials, and chrome steel beads were washed before use. To clean the XXTuff Reinforced vial, 1 mL MeOH was added to the vial and vortexed for 10 s. The solution was discarded, and the same process was repeated three times. Vials were then dried in a SpeedVac (Refrigerated CentriVap Concentrator, LABCONCO) for at least 1 h to remove residual solvent. To clean chrome steel beads, a clean glass beaker was filled with water and chrome steel beads were added into it. We gently shook the beaker for 30 s, and the wash solution was discarded. The process was repeated three times. The beads were then washed with isopropanol (IPA) using the same procedure three times. Finally, the washed chrome steel beads were dried in a 40°C Isotemp oven for 1 h. The dried clean beads were stored in a sealed glass container for future use. Detailed vial and bead cleaning can be found in Supplementary Texts 1, 2, respectively.

### Extraction of Short-Term Exposures

Short-term exposure chemicals were defined as the chemicals that are adsorbed on the surface of hair strands. To extract these metabolites, hair samples were mixed with 1 mL solvent mixture [H_2_O:MeOH:Acetone (1:1:1, v/v)] and shaken for 1 min on a shaker (Fisher Scientific). The solution was transferred out into an Eppendorf vial. The number of repeated washings were evaluated, and four repeated washings was considered optimal. The wash solution was dried using SpeedVac, reconstituted with 100 μL ACN:H_2_O (1:1, v/v) and transferred to a glass insert for LC-MS analysis to unbiasedly profile short-term exposure chemicals on hair.

### Extraction of Endogenous Metabolites and Long-Term Exposures

Endogenous metabolites and long-term exposure chemicals are embedded in hair strands. These chemicals can be extracted after homogenizing the hair strands to a powder. To improve homogenization efficiency, we first dried the hair samples using SpeedVac for 5 h. Long hair strands were cut short to ~1 cm pieces and added together with three cleaned chrome steel beads into a vial. The vials were put in a Mini-BeadBeater-24 (BioSpec, Bartlesville, Oklahoma) for homogenization for 30 s. Powdered hair was transferred into a clean Eppendorf vial for further analysis, during which the mass of transferred hair was recorded for sample normalization. One milliliter of MeOH was added to the powdered hair sample and sonicated in an ice bath (Branson CPX3800 Ultrasonic Cleaning Bath, Thermo Fisher Scientific) with 40 kHz rugged industrial transducers for 1 h. After that, the hair sample solution was centrifuged at 14,000 rpm to settle the insoluble particles, and the clear supernatant was transferred into a clean Eppendorf vial. The supernatant was dried in SpeedVac at room temperature. Finally, the dried sample was reconstituted in 100 μL ACN:H_2_O (1:1, v/v) and transferred to a glass insert for LC-MS analysis.

### LC-MS Analysis

Metabolite solutions of hair extract and hair wash were analyzed in an Agilent 1290 series ultra-high-performance liquid chromatography system (Agilent Technologies, Palo Alto, CA) coupled to a Bruker Impact II quadrupole time-of-flight (Q-TOF) mass spectrometer (Bruker Daltonics Billerica, MA). A Waters ACQUITY BEH-C18 column (100 × 1.0 mm, 1.7 μm particles) was used for reverse phase (RP) metabolite separation. LC-MS analysis was carried out in both positive and negative electrospray ionization (ESI) modes to achieve a comprehensive metabolome coverage.

For LC-MS in ESI positive mode analysis, mobile phase A was H_2_O with 0.1% formic acid (FA); mobile phase B was ACN with 0.1% FA. The LC gradient was as follows: 0 min, 95% A; 8 min, 75% A; 14 min, 30% A; 20 min, 5% A; 23 min, 5% A; 23.01 min, 95% A; 30 min, 95% A. The optimized injection volume was 10 μL, and the flow rate was 0.15 mL/min. The scan range of the mass spectrometer was 65–1,500 *m/z* with a data acquisition rate of 8 Hz. The capillary voltage was set at 4.50 kV (ESI+); the ion source temperature was set at 220°C; nebulizer and dry gas flow were 1.6 bar and 7.0 (L/min), respectively. All the optimization was done in ESI+ mode.

For LC-MS in ESI negative mode analysis, mobile phase A was H_2_O with 5% ACN and 10 mM ammonium acetate (adjusted to pH = 9.8 with ammonium hydroxide); mobile phase B was ACN with 5% H_2_O. The LC gradient was as follows: 0 min, 95% A; 8 min, 75%; 14 min, 30% A, 20 min, 5% A, 23 min, 5% A; 23.01 min, 95% A; 30 min, 95% A. The optimized injection volume was 2 μL, and the flow rate was 0.15 mL/min. The MS scan range was 65–1,500 *m/z* with a data acquisition rate of 8 Hz. The capillary voltage was set at 3.00 kV (ESI-); the ion source temperature was set at 220°C; nebulizer and dry gas flow were 1.6 bar and 7.0 (L/min), respectively.

### Data Processing

Metabolomic data was converted to .mzXML format using MSconvert (Adusumilli and Mallick, 2017). Raw LC-MS data are available on MetaboLights (ID: MTBLS2577). Raw LC-MS data were preprocessed using a recently published integrated feature extraction (Hu et al., 2019), which has better sensitivity in detecting low-abundant metabolic features. Detailed statistical analysis was performed using MetaboAnalyst (Xia et al., 2015) following the in-depth spectral annotation. Metabolite annotation was performed using dot-product based exact match against MoNA MS^2^ spectra library and McSearch (Xing et al., 2020) with one biotransformation reaction (level 2 annotation) (Schymanski et al., 2014). In the cases where multiple hits are returned in the search, we manually checked the results to see which one is more reasonable. Usually, the one with the highest matching score was picked. Classification of annotated metabolites was performed by searching the chemical classes in HMDB (Wishart et al., 2018). Detailed parameter settings for this study can be found in Supplementary Texts 3, 4.

### Biological Application

Hair samples after regular washing and after 48 h of environmental exposure (Supplementary Figure 4) were collected, respectively, from three female volunteers. These two sets of hair samples were compared to illustrate the environmental effects that are reflected in hair exposome. The three female volunteers selected are all in their twenties, have similar diet, long hair (25–30 cm), and natural black hair with no previous hair dyeing experiences. Three replicates were used for each condition. Hair extracts were also compared to illustrate the stability of the endogenous and long-term exposure metabolites that are firmly attached to the hair shaft. Our volcano plots with *p*-value threshold of 0.05 and fold change threshold of >1.5 or <0.67 show the number of significantly changed metabolic features for both hair wash and hair extract (before vs. after exposure).

## Results and Discussion

### Analytical Procedure

**Figure 1:**
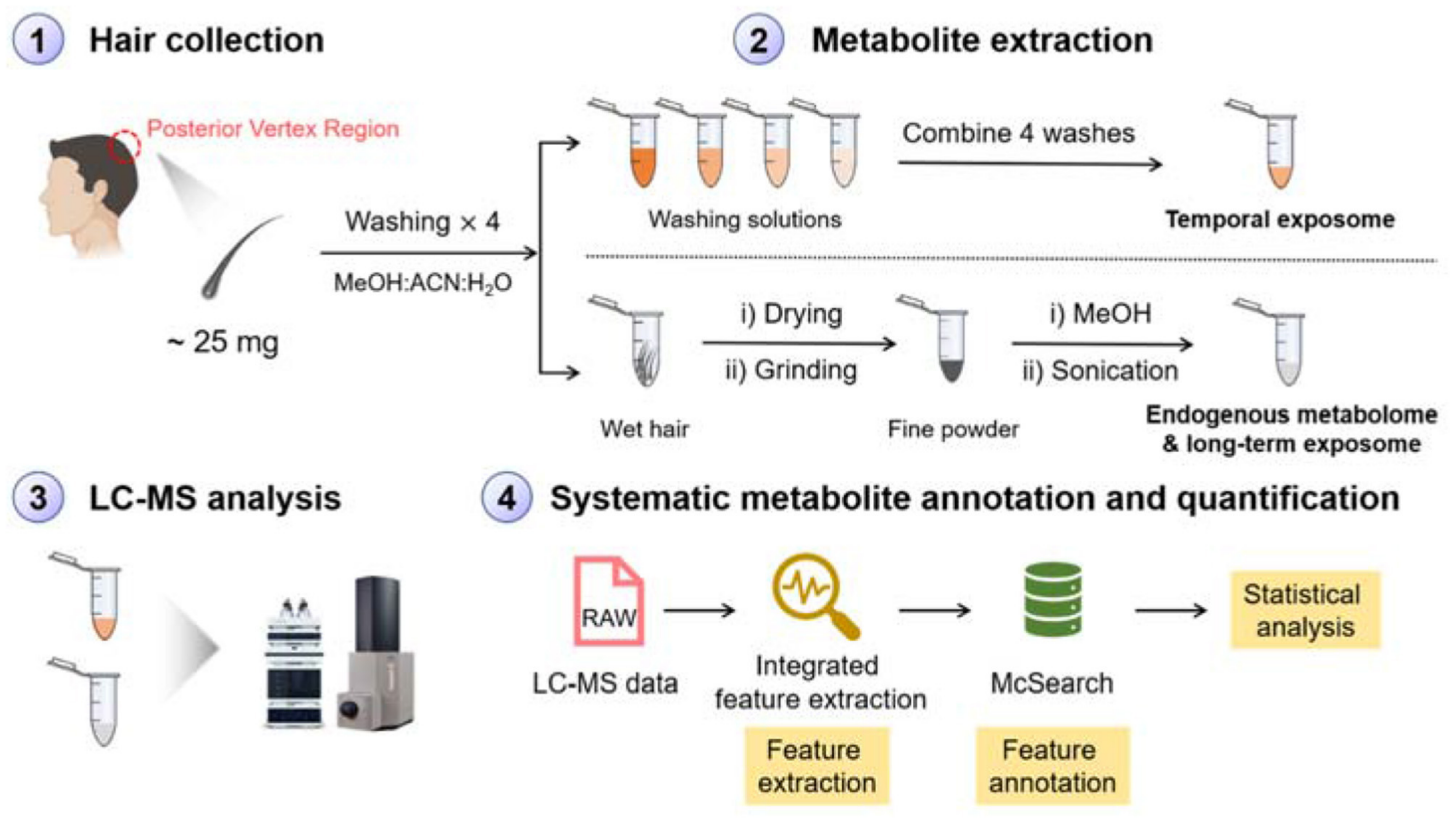
Schematic workflow of hair sample preparation, LC-MS analysis, data processing, and metabolite annotation for simultaneous profiling of endogenous metabolites, temporal, and long-term exposures.

Hair wash is usually applied to get rid of surface contaminants in the analysis of hair chemicals (Kintz et al., 2006). In our work, we argue that metabolites on the surface of hair can also be important for understanding temporal environmental exposure that are adsorbed on the surface of hair strands. These chemicals represent an individual's exposures on a daily basis and can be extracted using organic solvent. On the other hand, metabolites embedded in hair strands represent endogenous metabolites and long-term exposures. These metabolites can be extracted via conducting the homogenization and organic solvent extraction procedures (Eisenbeiss et al., 2019). To simultaneously detect both short- and long-term exposure chemicals, we designed a novel MS-based metabolomics workflow as shown in Figure 1. Photos of the equipment and hair before and after homogenization are shown in Supplementary Figure 1. Following metabolite extraction, we applied the LC-MS platform to profile metabolites in both ESI(+) and ESI(–) modes. The collected metabolomic data were further processed using recently developed feature extraction program targeting low-abundant metabolic features (Hu et al., 2019) and metabolite annotation software (Xing et al., 2020) aiming to annotate “unknown unknown” metabolites that are not archived in a spectral database. The annotated metabolic feature table can then be used for downstream data interpretation to understand how short- and long-term exposures as well as endogenous metabolites are changed over different exposure situations.

Hair samples from a female were used for the optimization of short-term metabolite extraction. Different conditions were validated on these hair samples to achieve optimal performance MeOH/H_2_O/ACE. The wash solution was analyzed using LC-MS in RP(+) mode.

**Figure 2:**
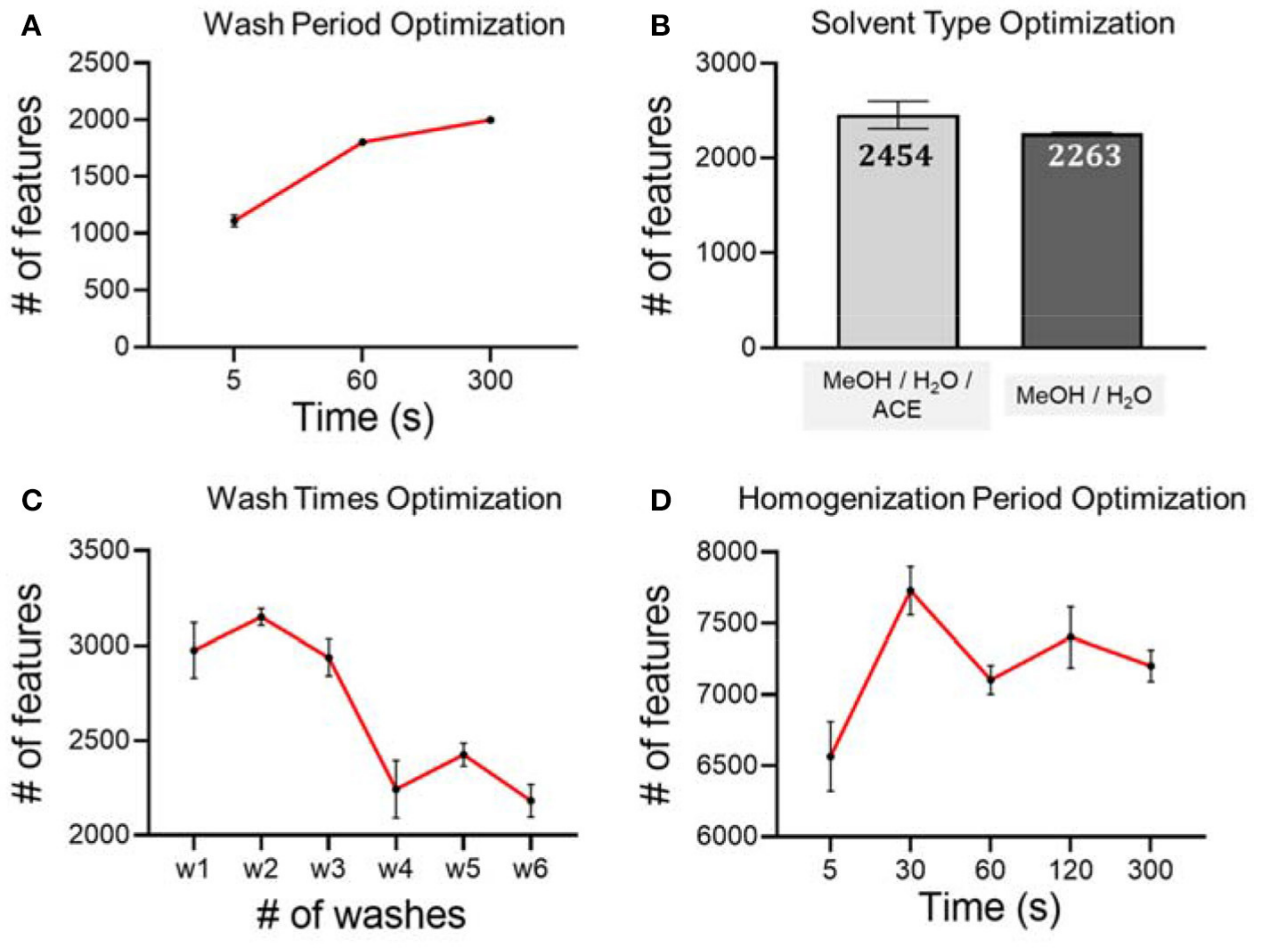
Optimization of experimental conditions using RP (+) mode and analytical triplicates. **(A)** Hair washing period; **(B)** hair washing solvent; **(C)** hair washing times; **(D)** hair homogenization period.

As seen in Figure 2A, we first tested on different hair wash durations. Our hypothesis is that a longer wash leads to more metabolites being washed off from hair strands. However, if the wash period is longer than necessary, it will slow down the experimental throughput without benefitting the analytical procedure. In this work, we tested three washing periods, including 5, 60, and 300 s. Considering the number of metabolic features and experimental throughput, we finally chose 300 s as the wash period. We then used the optimal wash period to carry forward and optimize the wash solvent type. We found that the number of metabolic features detected from the wash solution of MeOH/H_2_O/ACE is similar to the number of metabolites detected from MeOH/H_2_O, but slightly higher. Therefore, we used MeOH/H_2_O/ACE as a more efficient solvent in extracting exposure chemicals on hair surfaces for the following work (Figure 2B). Furthermore, we tested the number of washes that were needed to achieve complete removal of the adsorbed chemicals on hair samples using the pre-determined wash period and solvent. Washing a sample multiple times can definitely improve extraction efficiency as it has been demonstrated in the metabolite extraction in blood, urine and other biological samples (Wu and Li, 2013). In this work, we tested up to six washes and found that after four washes, the number of chemicals is significantly reduced (Figure 2C). Therefore, four washes were used in later experiments for the extraction of temporal hair metabolites. However, we also noticed that even after six washes, there is still a large number of metabolic features detected. Our results of high similarity are consistent with previous work showing that some metabolites are likely incorporated in inner hair compartments and are hardly deposited on the hair surface (Eisenbeiss et al., 2019).

Besides the optimization of short-term metabolite extraction, we also optimized the unbiased extraction of endogenous metabolites and long-term exposures from hair strands. Conventional hair sample digestion, including the use of strong acids, heat, or enzymatic treatment, leads to bias against labile chemicals as they are likely degraded during the digestion process. In order to preserve as many metabolites as possible, we chose to use mechanical force to pulverize the hair matrix for metabolite extraction. To achieve this goal, we incorporated bead beater-based homogenization using the hair samples from the same female. After trials and errors, we noticed that there are a few experimental details that need to be taken care of. First of all, if solvent was added prior to homogenization, hair strands become very hard to break down. Therefore, to improve downstream extraction efficiency, we mixed only steel beads with dried hair strands for homogenization. Next, we noticed that during the homogenization process, steel beads could scratch the inner surface of the vial. Direct addition of organic solvent for metabolite extraction can also extract contaminants from plastic vials, even though the suggested high-quality homogenization vials were used. This was proved by a comparison of method blank, which confirmed that this following solution is very effective at minimizing contamination during sample preparation with high extraction efficiency (Supplementary Figure 2). To solve this problem, we proposed to transfer the powdered hair to a clean Eppendorf vial before MeOH-based metabolite extraction. For the extraction of metabolites from hair strands, we tested different homogenization periods, including 5, 30, 60, 120, and 300 s. Our LC-MS analysis results suggest that homogenization for longer than 30 s does not lead to any further increase of metabolic features from hair extract solution (Figure 2D). Therefore, a homogenization period of 30 s is used as the optimized condition for the analytical procedure.

### Sample Normalization

Sample normalization is crucial in minimizing biological variation during quantitative metabolomics for obtaining meaningful biological interpretation. In comparative metabolomics, the same amount of each sample should be used to compare the metabolome changes resulting from two or multiple experimental conditions (Wu and Li, 2016). Regarding hair samples, an analytical challenge is that different hair samples have different amounts of water moisture (Barba et al., 2010). Because of this, direct comparison of hair samples by mass is not an accurate approach for sample normalization. To address this challenge, we proposed to dry hair samples using SpeedVac before measuring their mass. Hair samples used were from the same female volunteer, whose hair were used for hair wash and hair extract optimization experiments. A demonstration was provided in Supplementary Figure 3, in which we took a wet hair sample and dried it for two consecutive 5-h periods. Our results showed that the wet hair became completely dry after the first 5 h, and an additional 5 h of drying did not change its mass. Therefore, 5 h was used to dry hair samples before weighing for accurate sample normalization. The moisture of dry hair was found to be 7.9% (Supplementary Figure 3). In addition, since homogenized hair was transferred to another vial for metabolite extraction, the transferred hair powder was weighed again for the normalization of hair extraction samples.

**Figure 3:**
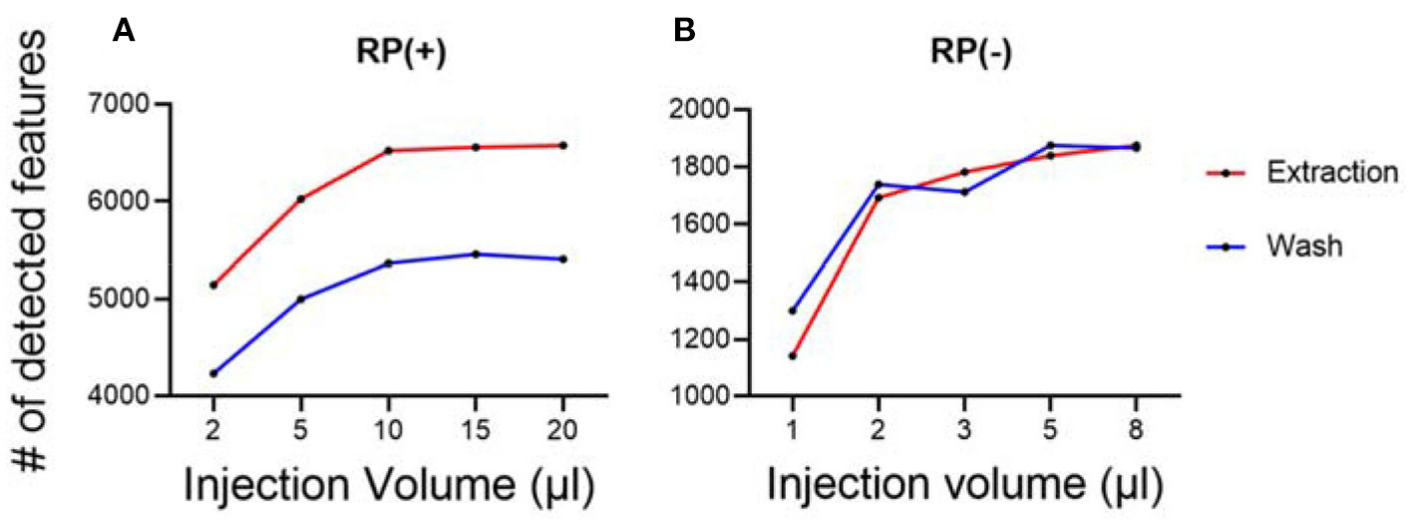
Optimization of sample injection amount for LC-MS analysis. **(A)** RP(+); **(B)** RP(–). Each condition has three replicates.

Besides sample normalization, the measured hair masses were also used to determine the optimal injection amount for LC-MS experiments. A series of injection volumes were tested and plotted against the number of detected metabolic features. The optimal sample injection volume was chosen where the maximum number of metabolic features was detected, and least peak saturation was observed. With this guidance, we tested using quality control samples and determined the optimal injection volumes for both hair wash and hair extract (Figure 3). The quality control samples were prepared by pulling equal aliquots from each sample in the group of extract or each sample in the group of wash. The optimized injection volumes allow the performance of untargeted hair metabolomics to be further improved with more detected metabolic features.

### Parallel Exposome and Metabolome Profiling

Hair wash and extract solutions were analyzed using LC-MS platform in both RP(+) and RP(–) modes for comprehensive chemical coverage. MS was operated in data-dependent acquisition (DDA) mode to acquire both quantitative information (i.e., MS^1^ data) for statistical analysis and structural information (i.e., MS^2^ data) for metabolite annotation (Guo and Huan, 2020). After data collection, raw LC-MS data was processed using a recently developed integrated feature extraction strategy to compensate the inability of conventional data processing software to extract low-abundant metabolic features (Hu et al., 2019). In brief, regular feature extraction was first performed using XCMS (Smith et al., 2006) followed by the extraction of low-abundant metabolic features using their high-quality MS^2^ spectra. Further data cleaning was performed to remove metabolic features with signal intensities lower than three-fold of their intensities in the method blank samples. A total of 7,591 and 7,415 were detected in RP(+) in the hair extract and wash solutions, respectively. In comparison, 2,414 and 2,169 metabolic features were detected in RP(–) for hair extract and wash solutions, respectively. Among all the detected metabolic features, 274 and 276 features were directly annotated using dot-product spectral matching against MoNA spectral library at a MS^2^ spectral similarity cutoff of 0.7 for hair wash and hair extract (Supplementary Table 1), respectively. Furthermore, an additional 3,356 and 3,079 features were annotated using McSearch, in which biotransformation and spectral similarity search is considered, for hair wash and extract, respectively (Supplementary Table 2).

**Figure 4:**
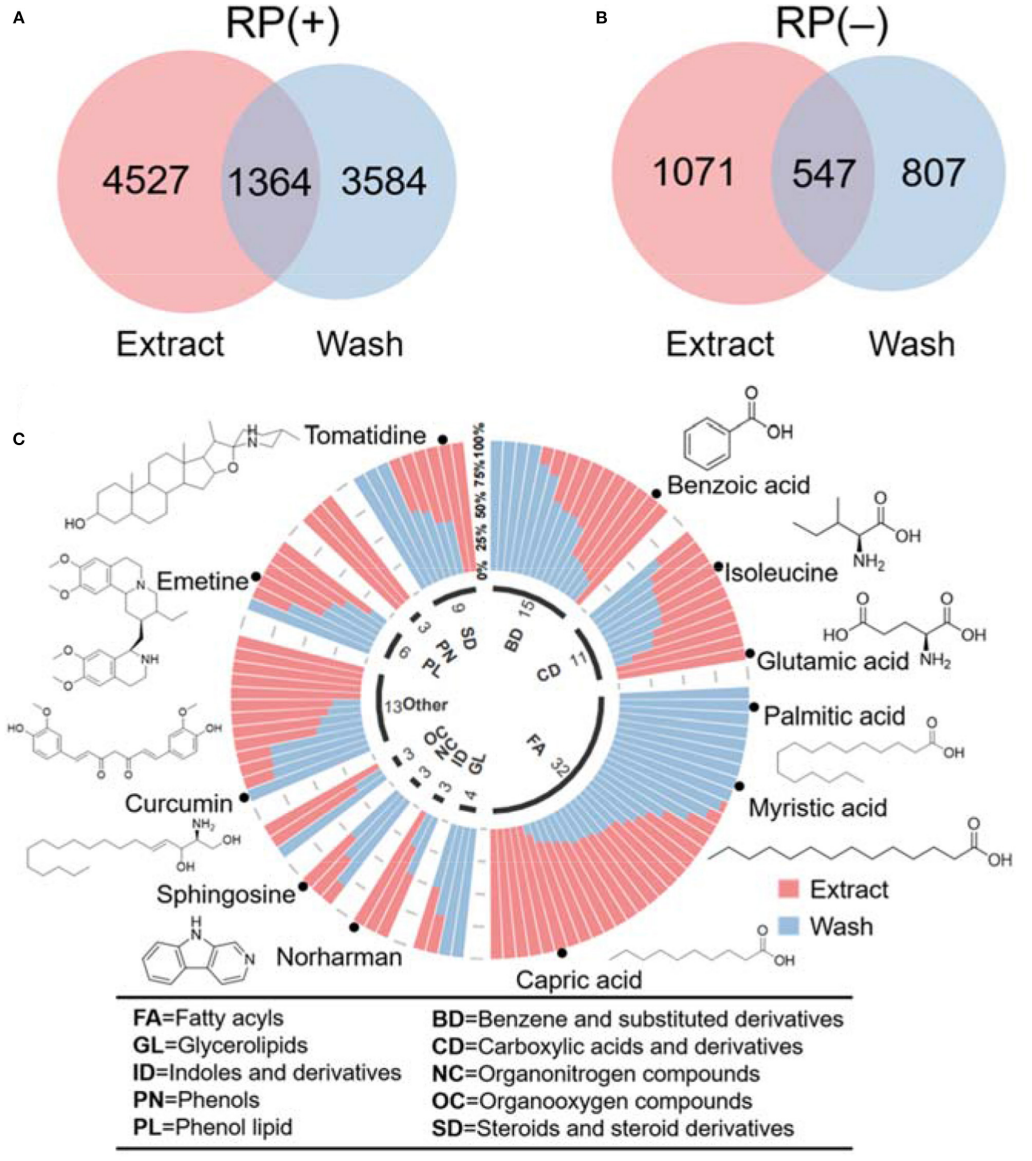
Venn diagrams illustrate the number of metabolomic features common and unique in hair wash and extract. **(A)** RP(+); **(B)** RP(–); **(C)** classification of annotated metabolites based on their chemical taxonomy in a circular stacked plot.

Using the profiling results, we further compared the unique and similar metabolic features between wash and extract results. Common metabolic features in wash and extract were identified using a retention time tolerance of 30 s and *m/z* of 0.01 ppm. The overlap results in Figures 4A,B indicate that there were a number of unique features presenting in both hair wash and hair extract along with a portion of features that appeared in both. We further selected the common metabolic features that were confidently annotated via MS^2^ spectral matching and compared their abundance in hair surface and hair strands. To make a fair comparison, we first calculated the unit intensity (counts/mg) of these metabolites in the wash and extract. Using the unit intensity values, we further calculated the proportion of a metabolite in extract using the average unit intensity of extract dividing by the sum of average unit intensity of hair extract and hair wash. When the intensity ratio of wash to extract was equal or >1.5, it was considered as dominant in wash; when the ratio found to be equal or <0.66, it was considered as dominant in extract. If the ratio was between 0.66 and 1.5, it was considered that metabolite could be found in equal or similar amount in both wash and extract. We categorized these metabolites based on their chemical class information in HMDB (Wishart et al., 2018; Supplementary Table 3). These data were also visualized in a circular stacked column plot (Figure 4C). Our results show that a significant number of metabolic features overlap between hair wash and hair extract. This agrees with previous work showing that some metabolites are likely incorporated in inner hair compartments and can only be partially washed off (Eisenbeiss et al., 2019).

### Proof-of-Principle Application

**Figure 5:**
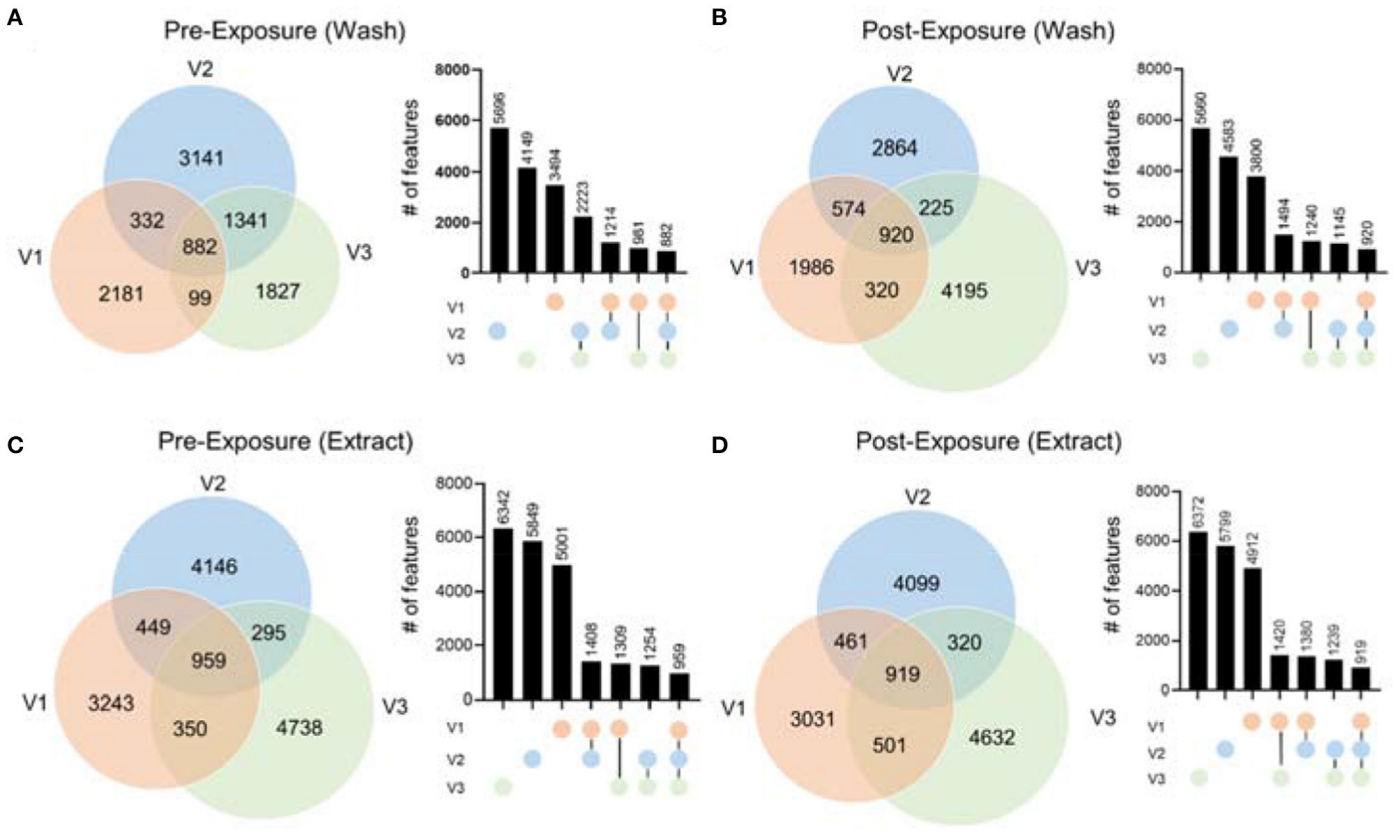
The comparison of metabolic features in hair extract and hair wash of the three volunteers before and after exposure in RP(+) mode. The Venn diagrams include **(A)** hair wash of pre-exposure; **(B)** hair wash of post-exposure; **(C)** hair extract of pre-exposure; **(D)** hair extract of post-exposure. Column plots next to each Venn diagrams illustrate the number of features in each area of the Venn diagram in the order from high to low.

To demonstrate the performance of our hair metabolomics workflow, we collected hair samples from three female volunteers right after they shampooed and rinsed their hair (i.e., before exposure), and re-collected after they were exposed to their own routine environment for 48 h (i.e., after exposure). Detailed physical activities during the 48 h were summarized in Supplementary Figure 4. Hair samples were collected and processed according to the optimized experimental procedure. Metabolomics data were collected and processed using the above-mentioned bioinformatic pipeline. From the metabolomics results, we first noticed that there were significant differences between the hair metabolomics of each individual. Figure 5 shows the Venn diagrams of hair wash and extract data, from RP(+), for the three individuals before and after exposure. The results for RP(–) mode are shown in Supplementary Figure 5. In both RP(+) and RP(–) results, while many metabolites are shared among the three individuals, we observed significant numbers of unique metabolites in both the hair wash and hair extract. Post-exposure (Figures 5B,D) lead to more unique metabolic features, which might be environmental exposures collected from the environment each individual was exposed to. Moreover, we noticed that regardless of before or after hair wash, significantly changed metabolites still remain, and its number is not affected by the exposure conditions. This agrees with the idea that human hair contains unique metabolic information that are characteristic of personal behavior and exposure (Liu et al., 2016).

**Figure 6:**
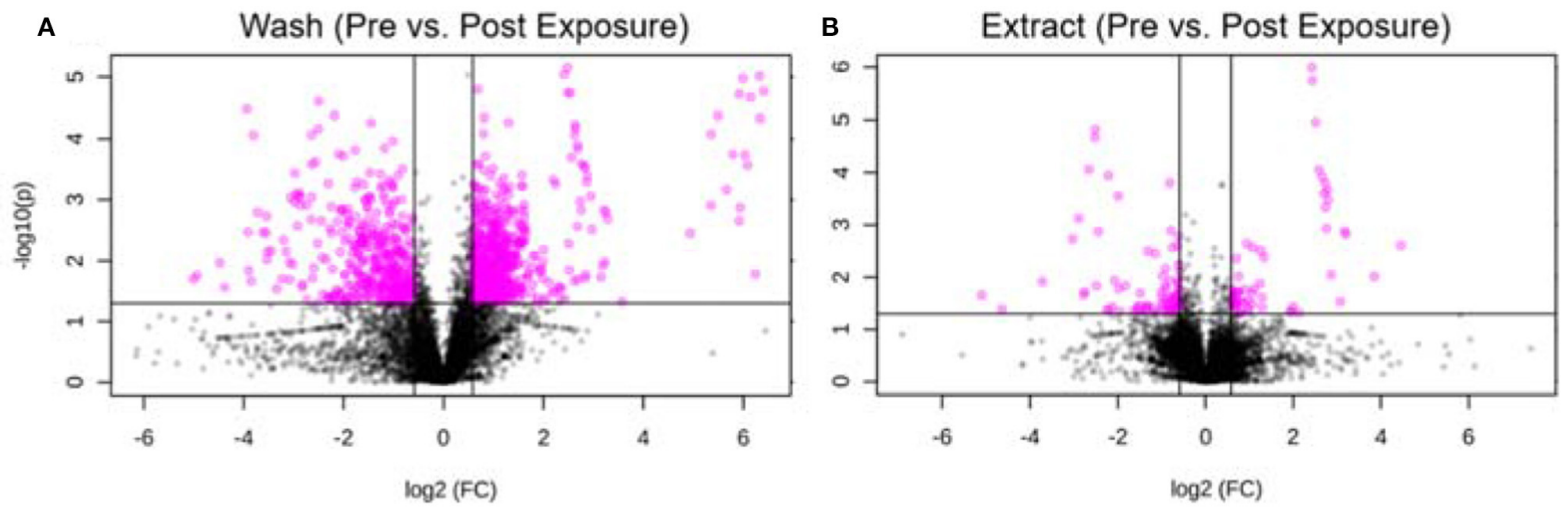
Volcano plot of significantly changed metabolic features. **(A)** Hair wash; **(B)** hair extract. The pink dots represent metabolic features that were significantly changed during the 2 days exposure. The black dots are features that have no significant difference before and after the 2 days exposure. The *p*-value threshold is 0.05 and the fold change threshold is >1.5 or <0.67. Six hundred and forty-five and eighty-nine metabolic features were significantly changed in the hair wash and hair extract solutions, respectively.

Furthermore, we took one individual and compared the metabolic changes before and after exposure for both hair wash and hair extract. Using thresholds of fold change (FC ≥1.5) and *p*-value (*p* ≤ 0.05), 645 and 89 metabolic features were significantly changed in the hair wash and hair extract solutions, respectively. Figures 6A,B show the visualization of these significantly changed metabolites using volcano plots for hair wash and hair extract, respectively. It can be clearly seen that the number of significantly changed metabolites in hair wash is much more than that in the hair extract. This is because the change of temporal exposures on hair surface is more dramatic than the change of endogenous metabolites or long-term exposures that are embedded in the hair strands over a 48-h exposure period. Among these significantly changed metabolic features, 372 (Supplementary Table 4) and 31 (Supplementary Table 5) metabolic features were annotated in hair wash and hair extract solutions using their experimental MS^2^ spectra and accurate precursor *m/z* values.

## Conclusion

Exposure to a wide range of xenobiotic chemicals plays a pivotal role in health and disease. Our development of a human hair metabolomics workflow resulted in a robust and sensitive analytical workflow to profile endogenous metabolites, short- and long-term exposure chemicals from human hair samples. With the help of state-of-the-art LC-MS instrumentation and bioinformatic programs, we detected over 5,000 features uniquely in hair extract, over 4,000 features uniquely in hair wash, and around 2,000 features that are common to both. The comprehensive characterization of endogenous and other exposure chemicals improves our understanding of global-scale hair metabolome. We believe that the approaches we provide in this study circumvented some major technical limitations commonly associated with hair exposome research. It could be easily applied to other hair metabolomics research to better understand exposome patterns via complete characterization of temporal exposure as well as endogenous metabolome of hair. Furthermore, the comprehensive definitions of exposome profile in the hair may improve our understanding of social activities reflected in hair samples and, ultimately, lead to future research possibilities of deciphering how environmental factors impact human health and disease.

## Data Availability Statement

The datasets presented in this study can be found in online repositories. The names of the repository/repositories and accession number(s) can be found in the article/Supplementary Material.

## Ethics Statement

The studies involving human participants were reviewed and approved by University of British Columbia. Written informed consent for participation was not required for this study in accordance with the national legislation and the institutional requirements.

## Author Contributions

YC and TH designed the experiment. YC and JG performed wet-lab sample preparation, LC-MS analysis, and data interpretation. SX provided help with chemical annotation. HY provided help with data visualization. YC, JG, and TH wrote the manuscript. All authors contributed to the article and approved the submitted version.

## Conflict of Interest

The authors declare that the research was conducted in the absence of any commercial or financial relationships that could be construed as a potential conflict of interest.
