# Supplementary material for: Global-Scale Metabolomic Profiling of Human Hair for Simultaneous Monitoring of Endogenous Metabolome, Short- and Long-Term Exposome
Source: Front Chem. 2021 May 12;9:674265. doi: 10.3389/fchem.2021.674265 (PMC8149753; doi:10.3389/fchem.2021.674265)
Supplement: Supplementary file 1 [file Data_Sheet_1.PDF]

Supplementary Information

Global-Scale Metabolomic Profiling of Human Hair for Simultaneous Monitoring of Endogenous Metabolites, Short- and Long-Term Exposome

Ying Chen[1], Jian Guo[1], Shipei Xing[1], Huaxu Yu[1], Tao Huan[1,*]

[1] Department of Chemistry, Faculty of Science, University of British Columbia, Vancouver Campus, 2036 Main Mall, Vancouver, V6T 1Z1, BC, Canada

[*] Author to whom correspondence should be addressed:

Dr. Tao Huan

Tel: (+1)-604-822-4891

E-mail: thuan@chem.ubc.ca

website: https://huan.chem.ubc.ca/

Table of Contents

Figure S-1. Wet lab demonstration.

Figure S-2. Contamination contributed from the scratched homogenization vial.

Figure S-3. Hair mass vs. drying period.

Figure S-4. Activity-log-48h of the three volunteers.

Figure S-5. Venn diagram of metabolic features detected in three individuals in RP(-) mode.

Text S-1. Vial cleaning.

Text S-2. Chrome steel bead cleaning.

Text S-3. Feature extraction parameter settings.

Text S-4. McSearch parameter settings.

Table S-1. Profile list for the identified metabolites in wash and extract.

Table S-2. Profile list for the McSearch identified metabolites in wash and extract.

Table S-3. Circular stacked column plot metabolites and their origins.

Table S-4. Significantly changed metabolites during the two days exposure for wash.

Table S-5. Significantly changed metabolites during the two days exposure for extract.

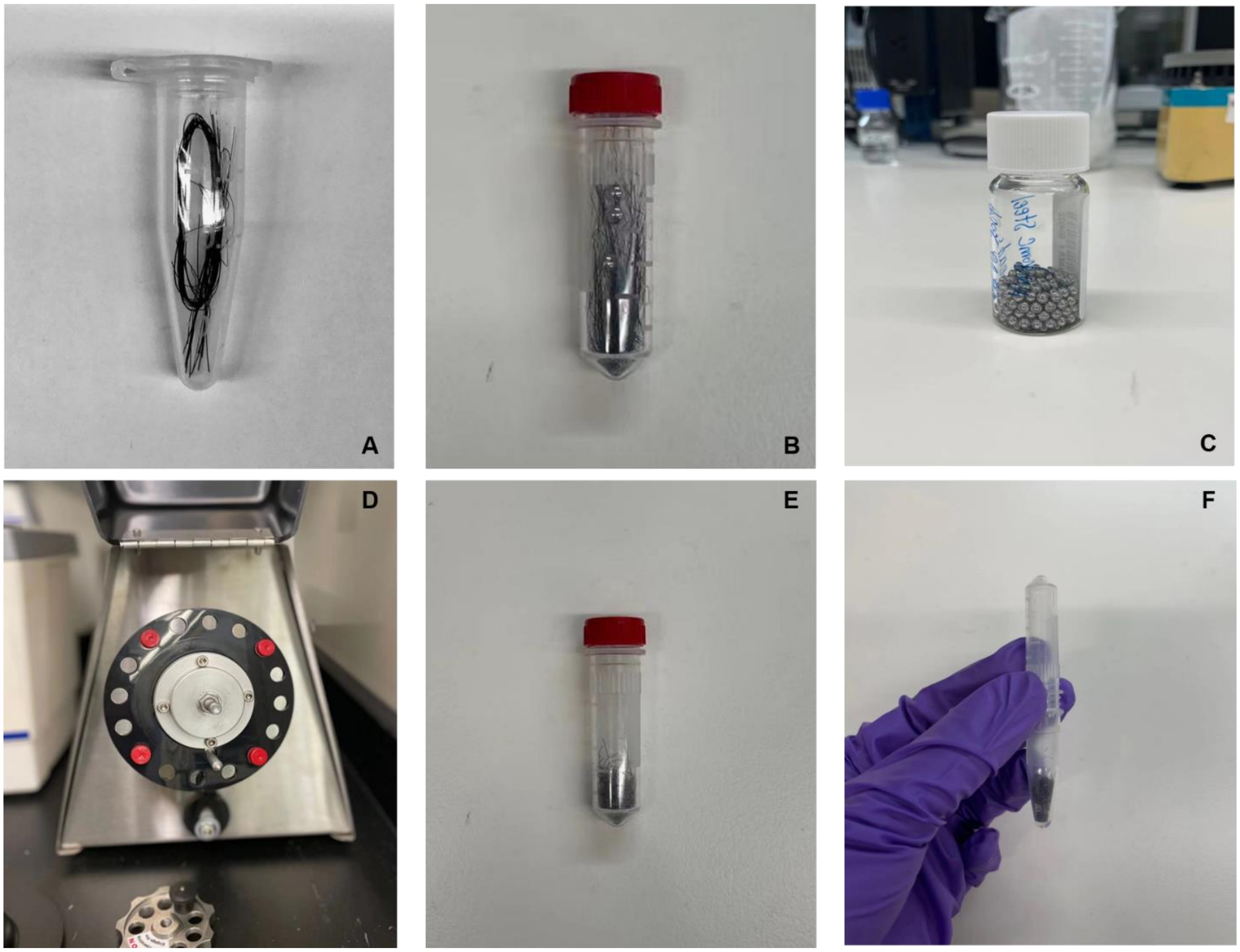

Figure S-1. Hair homogenization demonstration. **(A)** Hair sample was weighed into an Eppendorf vial and went through the wash procedures. **(B)** Washed hair was dried and transfer into a homogenization vial. **(C)** Three chrome steel beads were added into the homogenization vial. **(D)** Vials were placed into a homogenizer. **(E)** Beads were removed using a magnet. **(F)** The powdered hair sample was transferred into another Eppendorf vial by flipping the vial while the opening of the two vials were aligned.

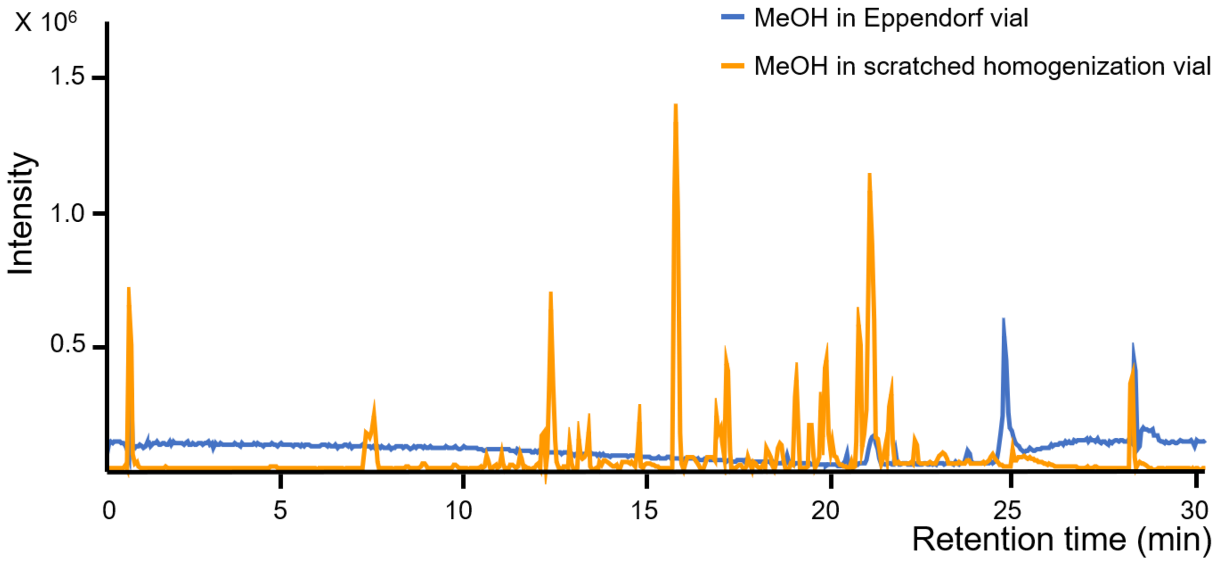

Figure S-2. Contamination contributed from the scratched homogenization vial. This experiment was done in RP (+) mode.

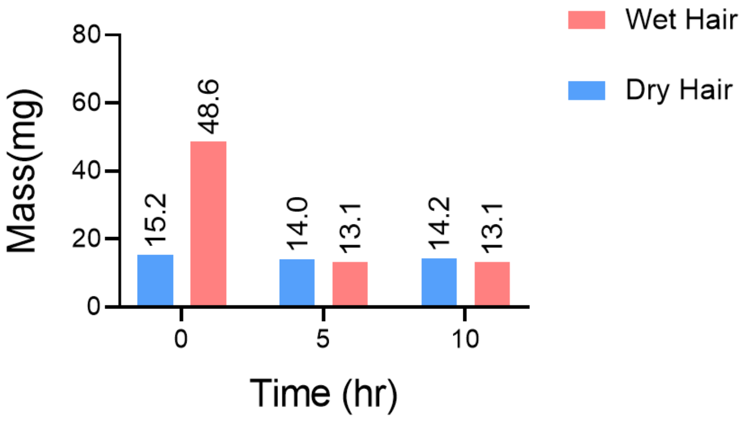

Figure S-3. Hair mass vs. drying period. Both dry and damp hair went through up to 10 hours of evaporation to determine the optimal evaporation period. The dry mass of the damp hair was measured and recorded. The moisture of the dry hair was 7.9% by weight.

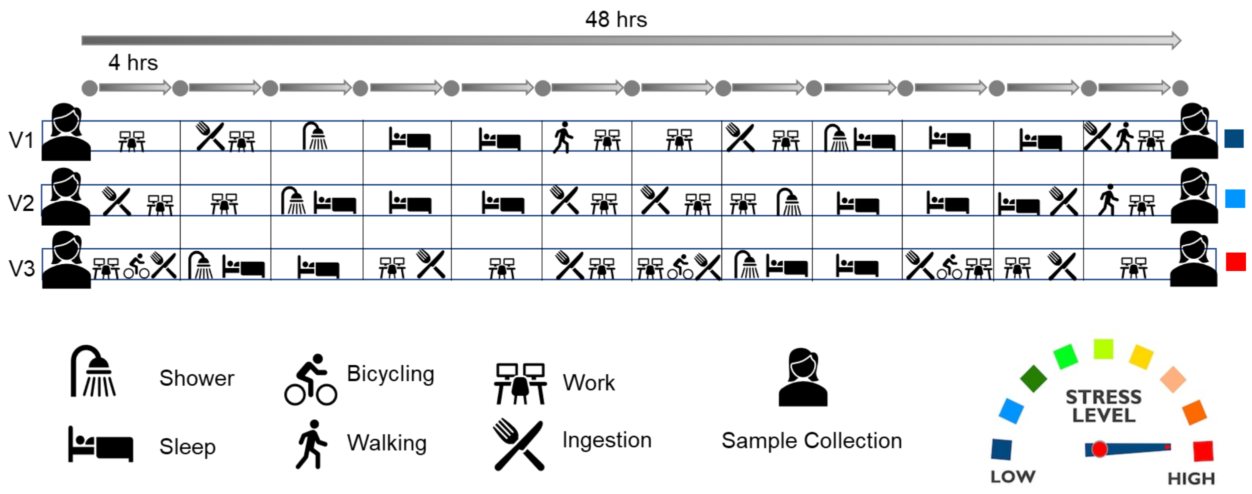

Figure S-4. Activity-log-48h of the three volunteers.

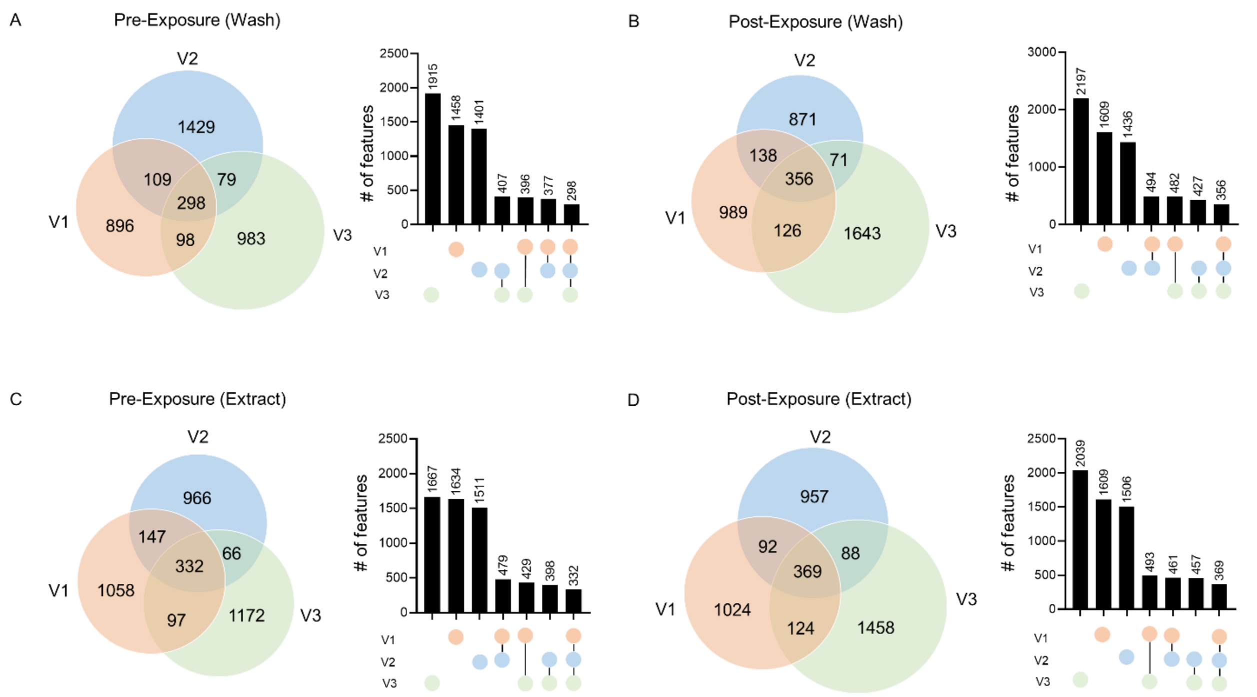

Figure S-5. Venn diagram of metabolic features detected in three individuals in RP(-) mode. (A) Hair wash of pre-exposure; (B) hair wash of post-exposure; (C) hair extract of pre-exposure; (D) hair extract of post-exposure.

Text S-1. Vial cleaning.

The XXTuff Reinforced vial is extremely durable and able to stand rigorous dry grinding with steel beads. It is made of a special high-grade polypropylene resin, and some trace materials are used in the production, such as organic mold releases. This could potentially cause some background noise in method blanks. Thus, cleaning vials before use is an important step in minimizing contamination. In brief, add 1 mL LC-MS grade methanol to the vial, vortex for 10 s and discard the solution. Repeat the same process three times. SpeedVac cleaned vials for at least 1 hr to evaporate all residual solvent. Clean, dry vials are ready to use. However, no organic solvent should be added to the XXTuff vial after the homogenization step since scratches on the inner surface will make the plasticizers to fall off, resulting in sample contamination and ion suppression.

Text S-2. Chrome steel bead cleaning.

Glass beads are normally used to homogenize biological samples. However, using glass beads to destroy hair samples is almost impossible because hair shaft is extremely tough and flexible. A stronger bead, chrome steel beads, was purchased from Biospec Products. We transferred the chrome steel beads into a clean beaker and added enough LC-MS grade water to cover all the beads. We gently shook the beaker for 30 s and discarded the wash solution. The same process was repeated three times. We then washed the beads with LC-MS grade isopropanol three times using the same method as above. The washed chrome steel beads were placed in 40 °C Isotemp oven for an hour for complete evaporation of residual solvent. Finally, the clean and dry beads were stored in a sealed glass container.

Text S-3. Parameter settings for feature extraction and dot product annotation.

Mass accuracy

Mass tolerance in finding level 3 features: 10 ppm

Mass tolerance in level 3 feature extraction: 0.05 Da

Retention time accuracy

Retention time tolerance in level 3 feature extraction: 60 s

Quantitative method

Peak height

Dot product annotation

MS^1 tolerance: 0.01 Da

MS^2 tolerance: 0.02 Da

Dot product threshold: 0.7

Match number threshold: 1

Library

MoNA Library:

https://mona.fiehnlab.ucdavis.edu/downloads/LC-MS/MS Positive Mode

https://mona.fiehnlab.ucdavis.edu/downloads/LC-MS/MS Negative Mode

Text S-4. McSearch parameter settings.

Mass accuracy

Mass tolerance for precursor search: 0.01 Da

Precursor searching mass range: 200 Da

Mass tolerance for HNL matching: 0.01 Da

Identification

Maximum number of HNL peaks used for spectral matching: 100

Minimum mass threshold for HNL vales: 36 Da

Library

MoNA Library:

https://mona.fiehnlab.ucdavis.edu/downloads/LC-MS/MS Positive Mode

https://mona.fiehnlab.ucdavis.edu/downloads/LC-MS/MS Negative Mode

R code

R script was downloaded: https://github.com/HuanLab/McSearch
